# Supplementary material for: Vibrational Spectroscopy as a Sensitive Probe for the Chemistry of Intra-Phase Bacterial Growth
Source: Sensors (Basel). 2020 Jun 18;20(12):3452. doi: 10.3390/s20123452 (PMC7348983; doi:10.3390/s20123452)
Supplement: Supplementary file 1 [file sensors-20-03452-s001.pdf]

SUPPLEMENTARY MATERIALS

Vibrational Spectroscopy as a Sensitive Probe for the Chemistry of Intra-Phase Bacterial Growth

Kamila Kochan [1,*], Elizabeth Lai [1], Zack Richardson [1], Cara Nethercott [2], Anton Y. Peleg [2,3], Philip Heraud [1,2] and Bayden R. Wood [1,*]

[1] Centre for Biospectroscopy and School of Chemistry, Clayton Campus, Monash University, Clayton, VIC 3800, Australia; ellai2@student.monash.edu (E.L.); zack.richardson@monash.edu (Z.R.); phil.heraud@monash.edu (P.H.)

[2] Infection and Immunity Program, Monash Biomedicine Discovery Institute and Department of Microbiology, Clayton Campus, Monash University, Clayton, VIC 3800, Australia; cara.nethercott@gmail.com (C.N.); anton.peleg@monash.edu (A.Y.P.)

[3] Department of Infectious Diseases, The Alfred Hospital and Central Clinical School, Monash University, Melbourne, VIC 3004, Australia

***** Correspondence: kamila.kochan@monash.edu (K.K.); bayden.wood@monash.edu (B.R.W.)

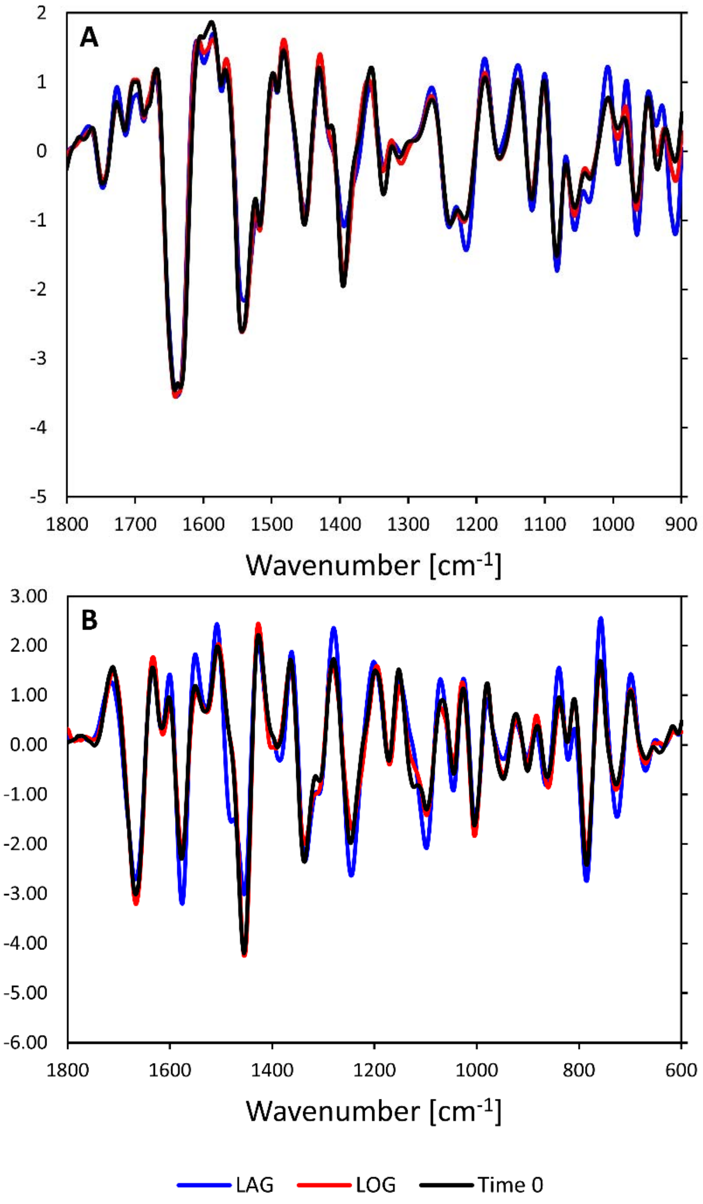

Figure S1. Comparison of IR (ATR) and Raman spectra recorded for bacteria in lag and log phases and at the beginning of the experiment (time 0). Average second derivatives of ATR spectra in the range of 1800–900 cm^{-1} are presented in (A) and average 2nd derivatives of Raman spectra in the range of 1800–600 cm^{-1} are shown in (B). Color-coding is given at the bottom of the Figure for both (A) and (B).
